# Biventricular Pulsus Alternans

**DOI:** 10.4061/2009/703793

**Published:** 2009-09-24

**Authors:** Param Vidwan, George A. Stouffer

**Affiliations:** Division of Cardiology, University of North Carolina, Chapel Hill, NC 27599-7075, USA

## Abstract

Pulsus alternans is a rare hemodynamic condition characterized by beat-to-beat variability in systolic pressure. It is attributed to variations in stroke volume with alternate cardiac cycles and is typically seen in patients with advanced myopathic conditions. Left ventricular pulsus alternans is rare, and right ventricular pulsus alternans is even less common. There are only a few reports of biventricular pulsus alternans. We report the case of a 62-year-old female with a recent anterior wall myocardial infarction who had biventricular pulsus alternans at the time of cardiac catheterization.

## 1. Case Report

A 62-year-old female with diabetes mellitus and hypertension developed what she thought was the “flu” associated with orthopnea, shortness of breath, and chest pressure. She presented to her local physician 3 weeks later when the symptoms persisted and were accompanied by a 15–20 pound weight gain. Her BNP was markedly elevated and a chest X-ray showed cardiomegaly with bilateral pleural effusion. ECG and echocardiogram were consistent with a recent anterior wall myocardial infarction.

**Figure 1:**
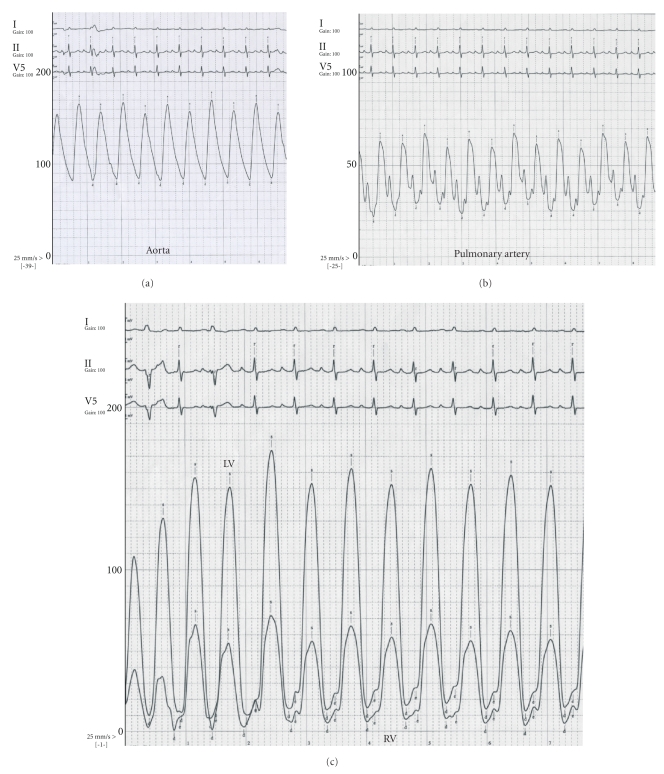
Pulsus alternans—beat-to-beat variation in systolic pressure is evident in aorta (a), pulmonary artery (b), and left and right ventricles (c).

Cardiac catheterization after a 15 pound diuresis showed severely reduced left ventricular systolic function with an ejection fraction of 31%. Left heart filling pressures were elevated with a left ventricular end diastolic pressure of 26 mmHg and pulmonary capillary wedge pressure of 28 mmHg. She had severe disease of the mid left anterior descending coronary artery which was treated with placement of a drug-eluting stent. During the procedure, the patient had beat-to-beat alterations evident in aortic, pulmonary artery, left ventricular, and right ventricular pressures (Figure 1) in the absence of RR cycle length variation which is diagnostic of pulsus alternans.

In the nine months since her cardiac catheterization, she has had no recurrent heart failure, angina, or myocardial infarction. She remains active and has NYHA class II symptoms on a medical regimen that includes metoprolol (25 mg BID), quinapril (40 mg qD), and furosemide (20 mg qD).

## 2. Discussion

Pulsus alternans was originally described more than 100 years ago. It is a rare finding in the left ventricle and even less common in the right ventricle. The cause of pulsus alternans has not been clearly delineated, and there is some speculation that different mechanisms may play a role dependent on the patient. One proposed mechanism is beat-to-beat alternations in stroke volume due to variation in preload and contractility. Impaired systolic contraction of a failing ventricle reduces stroke volume which then results in elevated end diastolic volume for the next contraction. Elevated left ventricular stretch results in increased stroke volume and therefore increased systolic pressure on the next beat [1]. Another proposed mechanism for the physiology of pulsus alternans involves abnormal calcium handling by cardiac myocytes [2]. In support of this hypothesis, cardiac-specific overexpression of calsequestrin, a major storage protein for calcium in the sarcoplasmic reticulum, resulted in significant decreases in contractile parameters and intracellular Ca^++^ transients and the development of pulsus alternans in a mouse model [3].

Severe left ventricular systolic dysfunction is the primary cause of pulsus alternans but other causes have been reported in humans including aortic stenosis [4, 5], hypertrophic obstructive cardiomyopathy [6], mitral stenosis [7], prosthetic valve dysfunction [8], and during dobutamine infusion [9, 10]. Right ventricular pulsus alternans has been reported in cases with severe right ventricular failure, primary pulmonary hypertension, prosthetic mitral valve thrombosis, diastolic or systolic left ventricular dysfunction, reactive airway disease, pulmonary embolus, and mitral stenosis [11–13]. Biventricular pulsus alternans has been described in a patient with severe left ventricular dysfunction and left anterior descending coronary artery disease [14], similar to the current case.
